# Supplementary material for: ER calcium stores contribute to glucose-induced Ca2+ waves and intercellular connectivity in mouse pancreatic islets
Source: Cell Calcium. Author manuscript; Available in PMC 2026 Aug 10. (PMC13456486; doi:10.1016/j.ceca.2025.103093)
Supplement: Supplementary file 1 [file NIHMS2187478-supplement-MMC15.pdf]

Supplementary figures

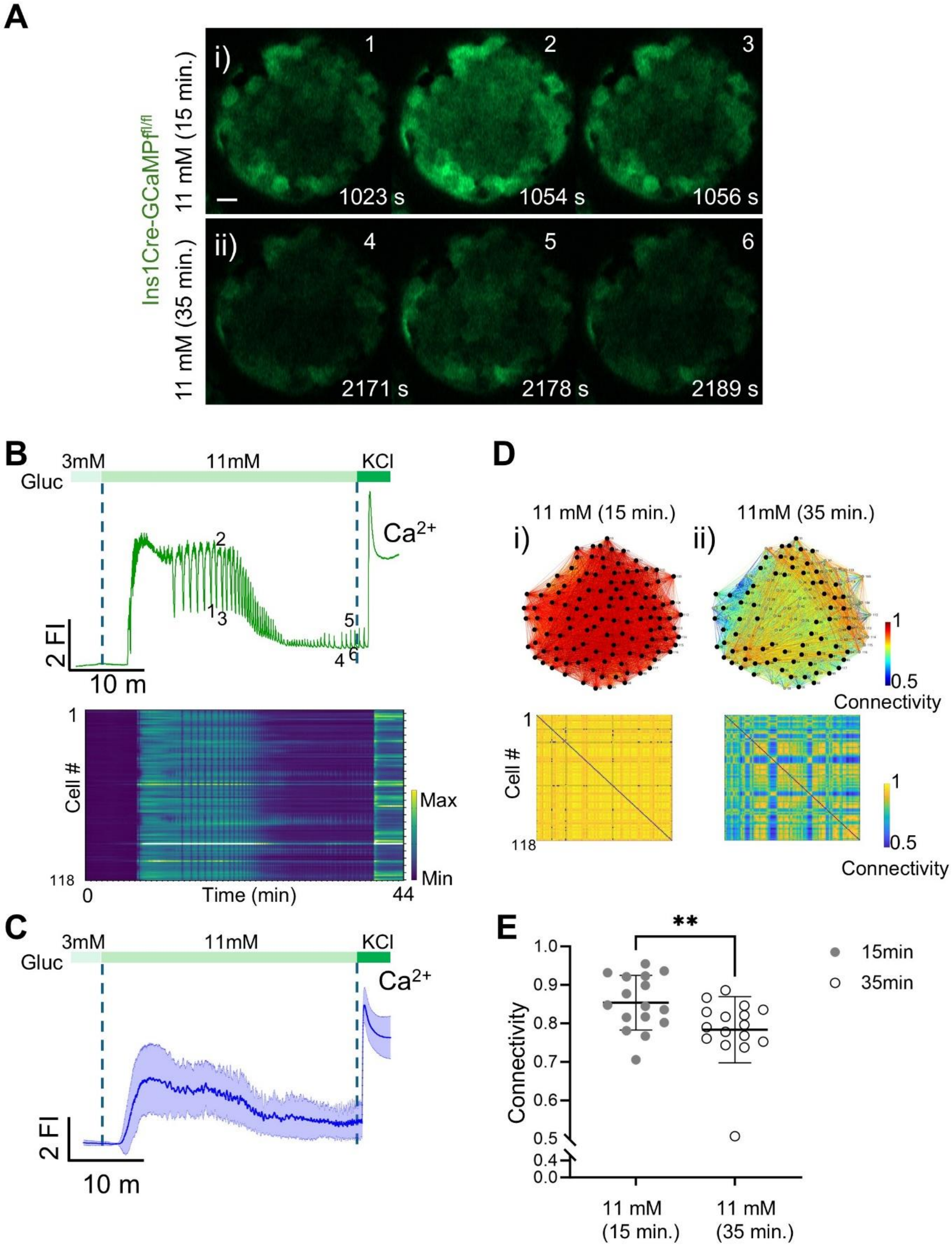

Supplementary Figure 1. Islet calcium dynamics exposed to constant 11mM. A) Series of confocal calcium imaging of pancreatic islet showing GCaMP6f signal specifically in beta-cells at the indicated time points (in sec.) during calcium waves oscillations at sustained 11 mM during **i)** the first 15min. and **ii)** 35min. Scale bar = 25 µm. **B)** Fluorescent trace of the GCaMP6 signal from the islet shown in **(A)** demonstrating calcium influx during the glucose ramp (3 mM, 11 mM) and membrane depolarization induced by 40 mM KCl. The raster plot corresponds to the signal from individual cells. **C)** Average calcium fluorescent traces from islets exposed to a glucose ramp (3 mM, 11 mM) and membrane depolarization by 40 mM KCl (n=3 WT 39-islets). The blue line shows the average GCaMP6f fluorescent and the light-blue the standard deviations from the imaged islets. **D)** Connectivity map and matrix of the islet shown in **(A)** from the **i)** first 15min. and **ii)** 35min. A connectivity of 1 indicates perfect coordination and 0.5 means that 50% of their active time a cell pair is coordinated. **E)** Connectivity from individual islets n=4 WT 16-islets (unpaired 2-tailed Student's t-test. ns= not significant).

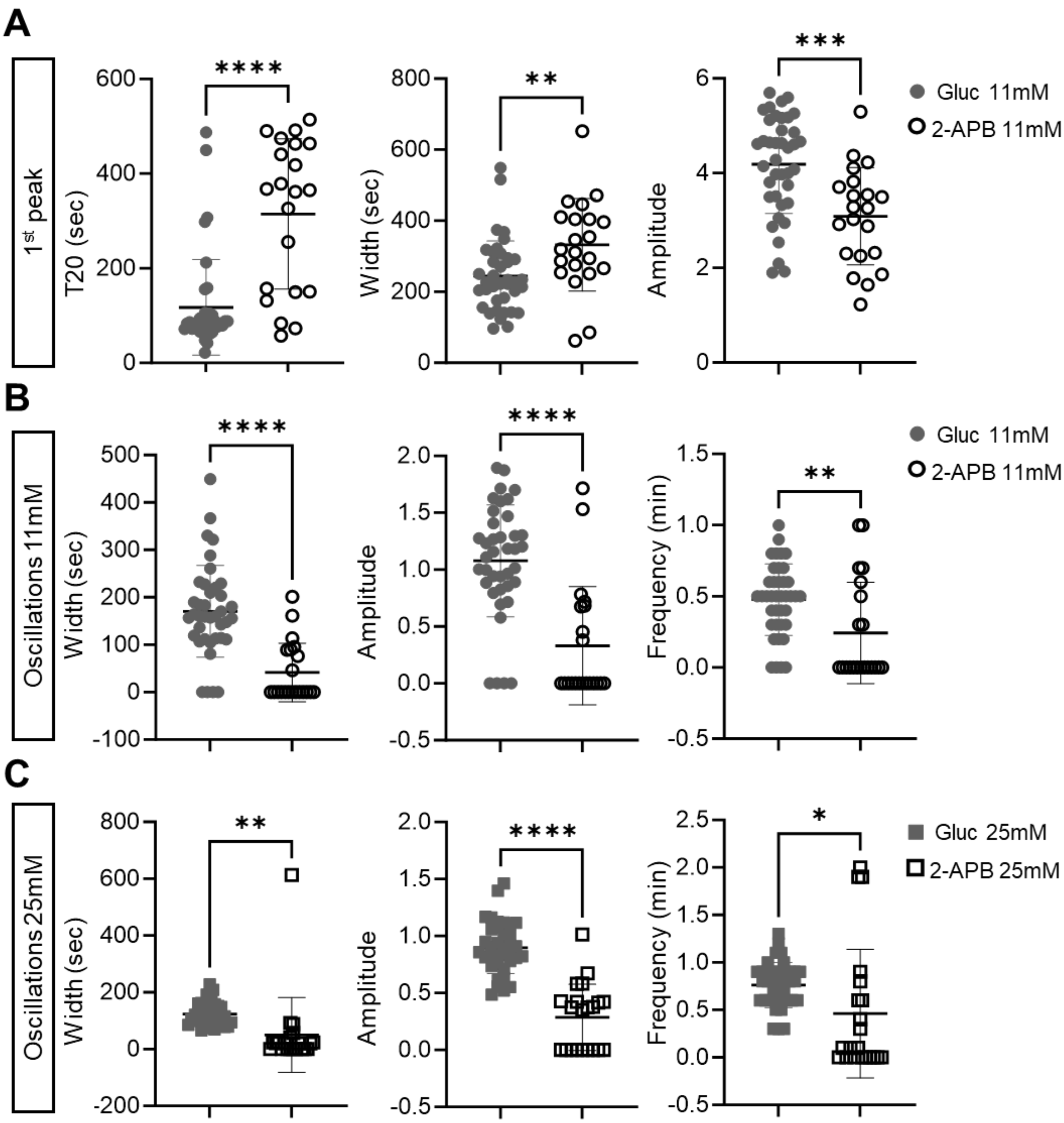

Supplementary Figure 2. IP_3R inhibition by 2-APB impairs Ca^{2+} dynamics. A) First-peak T_{20} is the time taken from the glucose addition in the media to present an increase of 20% over the baseline. The amplitude of the 1st peak is defined as the maximum value recorder during the 1st increase. The peak width is defined as the time spent with an intensity >20% over the base line. The graphs comparing the T_{20}, peak width and peak amplitude between glucose and 2-APB exposed islets. **B)** The frequency analysis at 11mM and 25mM covering the last 10 min of each section. Average peak width, peak amplitude and peak frequency quantifications of islets incubated with glucose and 2-APB at 11mM. **C)** Average peak width, peak amplitude and peak frequency quantifications of islets incubated with glucose and 2-APB at 25mM. Each symbol represents an islet (2-way paired ANOVA, Tukey's correction *$P \leq .05$, **$P \leq .01$, ****$P \leq .0001$, ns, not significant; n=3 WT 39-islets for glucose and n=3 WT 21-islets for 2-APB. Data are mean ± SD.)

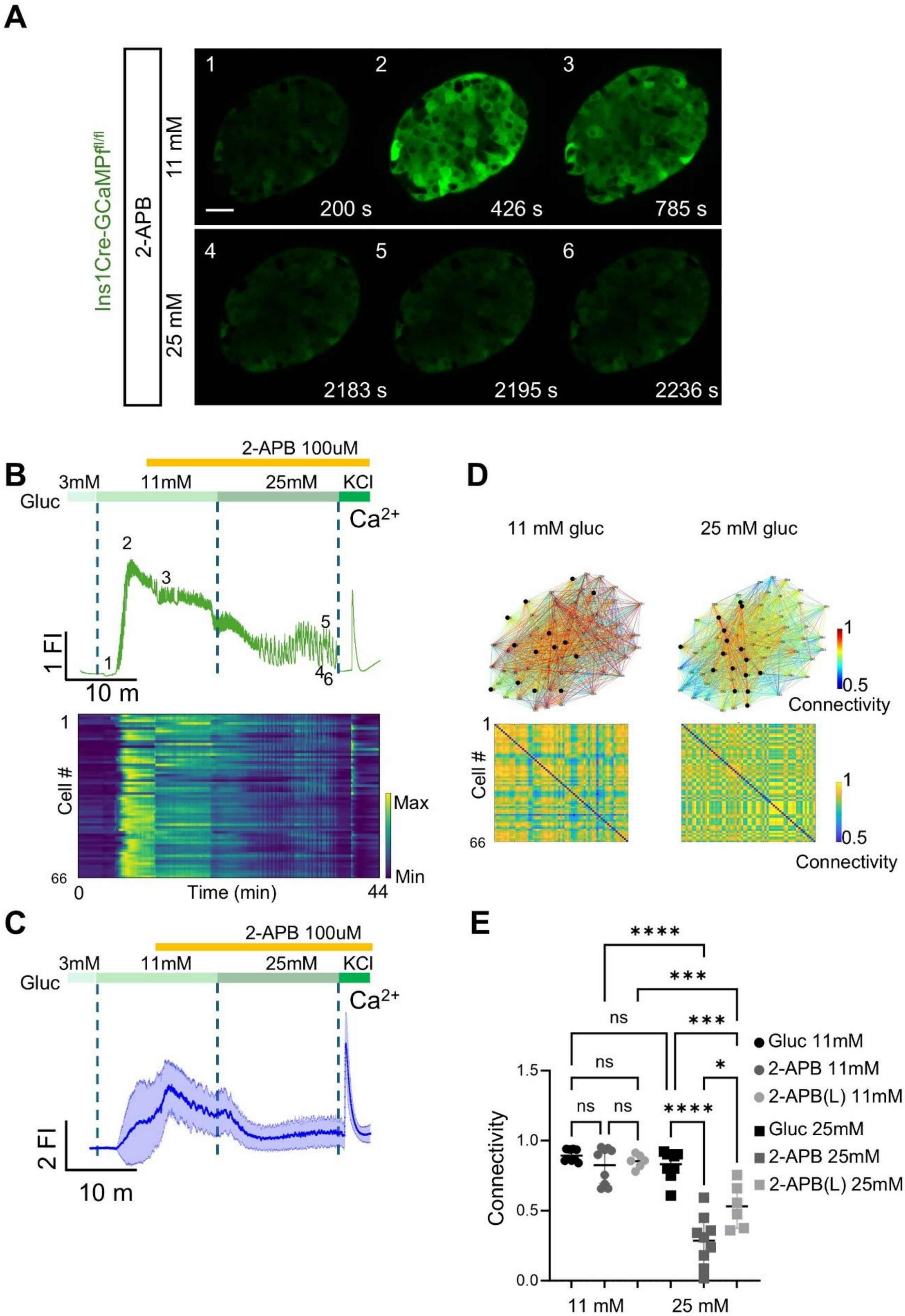
A
Ins1Cre-GCaMP$^{fl/fl}$
2-APB
11 mM
25 mM
1
2
3
200 s
426 s
785 s
4
5
6
2183 s
2195 s
2236 s
B
2-APB 100uM
3mM
11mM
25mM
KCl
Gluc
Ca^{2+}
1 FI
10 m
Cell #
Max
Min
Time (min)
0
44
66
C
2-APB 100uM
3mM
11mM
25mM
KCl
Gluc
Ca^{2+}
2 FI
10 m
D
11 mM gluc
25 mM gluc
Connectivity
Cell #
Connectivity
E
Connectivity
11 mM
25 mM
Gluc 11mM
2-APB 11mM
2-APB(L) 11mM
Gluc 25mM
2-APB 25mM
2-APB(L) 25mM
****
***
ns
***
ns
*
ns
ns
****

Supplementary Figure 3. Inhibition of IP_3R by 2-APB impairs glucose-induced intracellular Ca^{2+} dynamics and connectivity. A) Series of confocal calcium imaging of pancreatic islet at the indicated time points (in sec.), exposed to the IP3-inhibitor 2-Aminoethoxydiphenyl borate (2-APB) at **i)** 11 mM and **ii)** 25 mM glucose. Scale bar = 25 µm. The 2-APB was delivered after 7.5 min. of glucose increase from 3mM to 11mM. **B)** Fluorescent trace of the GCaMP6 signal from the islet shown in **(A)** during the glucose ramp (3 mM, 11 mM, 25 mM) and membrane depolarization by 40 mM KCl. The raster plot corresponds to the signal from individual cells. **C)** Average calcium fluorescent traces from islets exposed to 2-APB during a glucose ramp (3 mM, 11 mM, 25 mM) and membrane depolarization by 40 mM KCl. The blue line shows the average GCaMP6f fluorescent and the light-blue the standard deviations from the imaged islets (n=4 WT 103-islets). **D)** Connectivity map and matrix of the islet shown in **(A)** at **i)** 11 mM and **ii)** 25 mM glucose. **E)** Connectivity from individual islets glucose, 2-APB from the beginning of the recording or later addition 2-APB(L) (7.5m after glucose increase from 3mM to 11mM glucose). (2-way paired ANOVA, Tukey's correction $*P \leq .05$, $***P \leq .001$, $****P \leq .0001$, ns, not significant; glucose n=4 WT 9-islets; 2-APB n=4 WT 9-islets; 2-APB (Late) n=3 WT 6-islets)

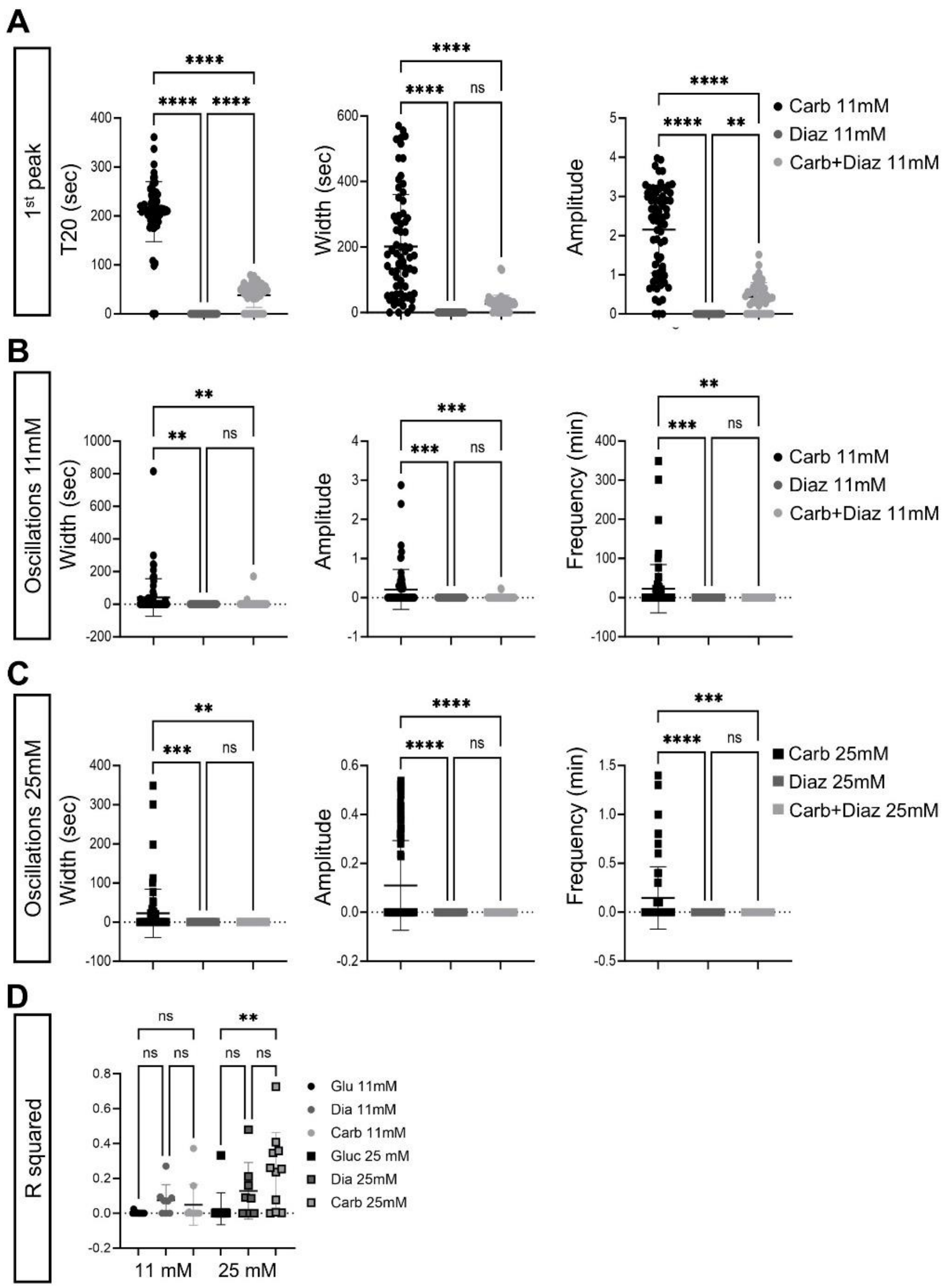

Supplementary Figure 4. Muscarine agonism by Carbachol impairs Ca^{2+} dynamics. A) The graphs comparing the T_{20}, peak width and peak amplitude between carbachol, diazoxide and carbachol+diazoxide exposed islets of the first-peak. **B-C)** The frequency analysis at 11mM and 25mM covering the last 10 min of each section. Average peak width, peak amplitude and peak frequency quantifications of islets incubated with carbachol, diazoxide and carbachol+diazoxide at 11mM. (2-way paired ANOVA, Tukey's correction **P* ≤ .05, ***P* ≤ .01, ****P* ≤ .001, *****P* ≤ .0001, ns, not significant; n=3 WT 71-islets for carbachol, n=3 WT 45-islets for diazoxide and n=3 WT 47-islets from diazoxide+carbachol) **D)** R-squared quantifications of the probability to present connections vs the connectivity for individual cells (2-way paired ANOVA, Tukey's correction ***P* ≤ .01, ns, not significant; n=3 WT 13-islets for carbachol, n=3 WT 9-islets for diazoxide and n=3 WT 11-islets from diazoxide+carbachol. Data are mean ± SD.)

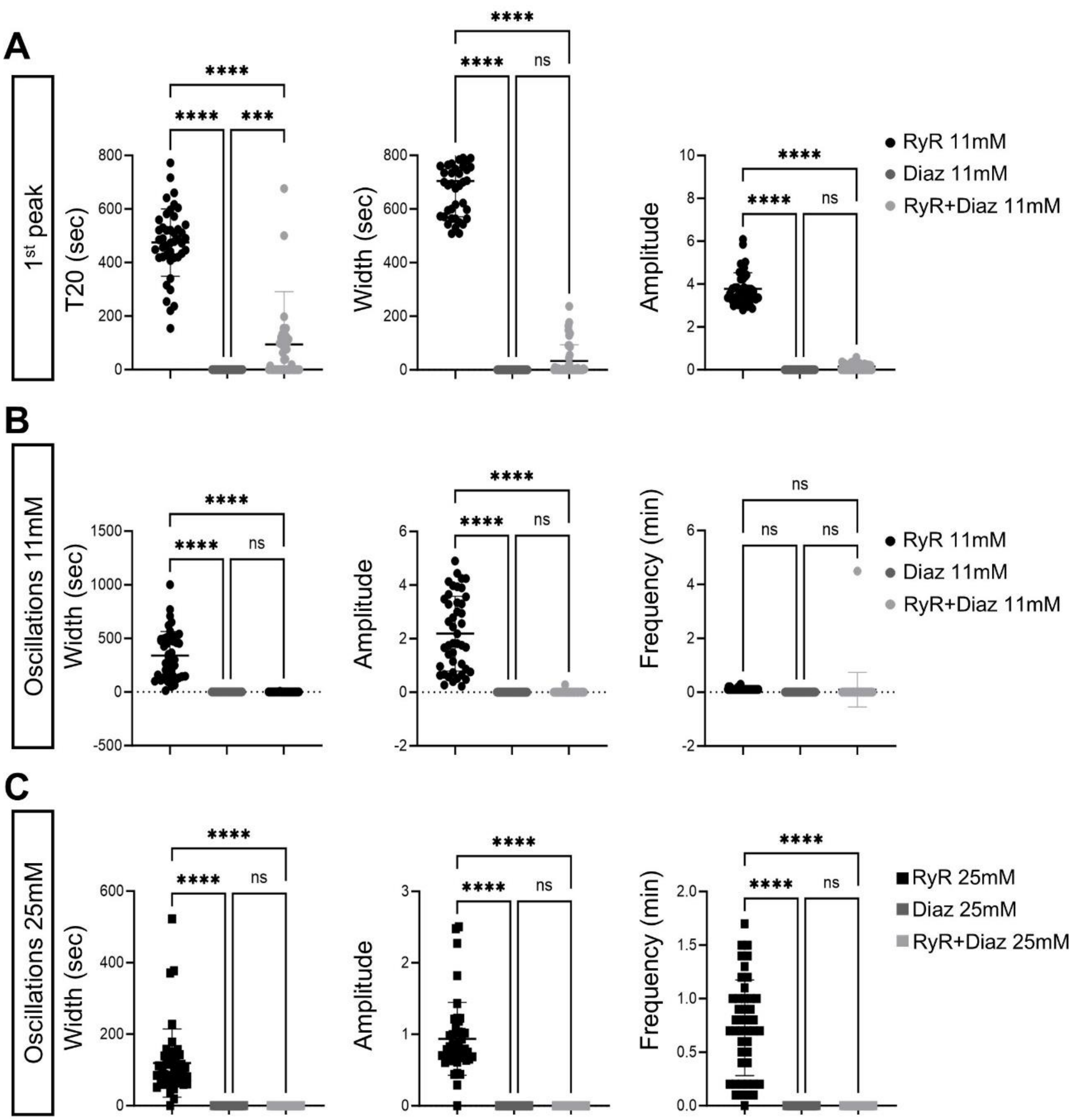

Supplementary Figure 5. Ryanodine receptor agonism increases frequency and induces calcium transients under hyperpolarizing conditions. A) The graphs show the T_{20}, peak width and peak amplitude between ryanodine, diazoxide and acute ryanodine+diazoxide exposed islets. **B)** The frequency analysis at 11mM and 25mM covering the last 10 min of each section. Average peak width, peak amplitude and peak frequency quantifications of islets incubated with ryanodine, diazoxide and acute ryanodine+diazoxide at 11mM. **C)** Average peak width, peak amplitude and peak frequency quantifications of islets incubated with ryanodine, diazoxide and acute ryanodine+diazoxide at 25mM. Each symbol represents an islet (2-way paired ANOVA, Tukey's correction $*P \leq .05$, $**P \leq .01$, $***P \leq .001$, $****P \leq .0001$, ns, not significant; n=3 WT 44-islets for ryanodine, n=3 WT 45-islets diazoxide and acute n=3 WT 49-islets ryanodine+diazoxide. Data are mean ± SD.)

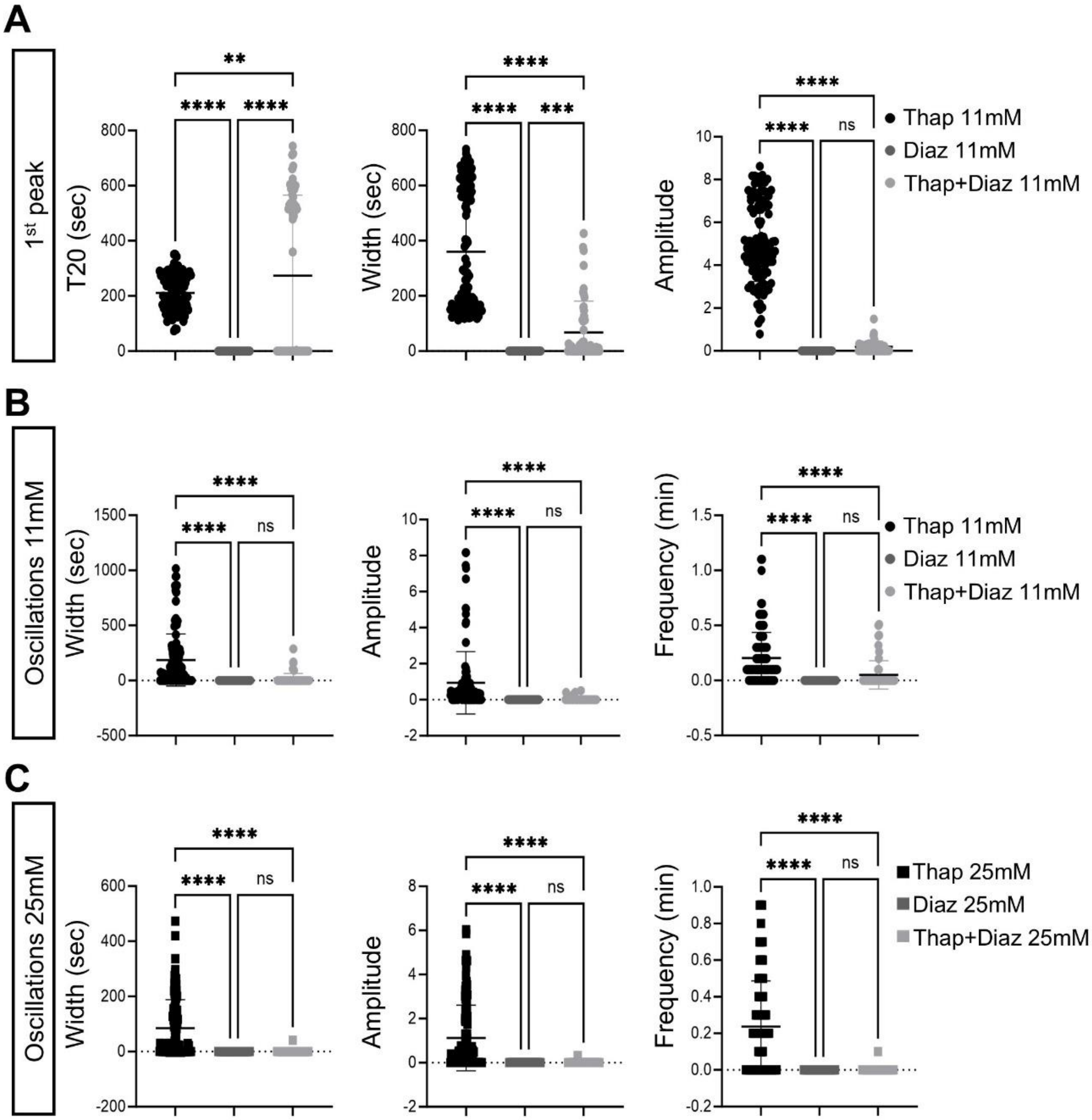

Supplementary Figure 6. Inhibition of SERCA2 by thapsigargin impairs Ca^{2+} dynamics and connectivity. A) The graphs show the T_{20}, peak width and peak amplitude between 1h thapsigargin, diazoxide and acute thapsigargin+diazoxide exposed islets. **B)** The frequency analysis at 11mM and 25mM covering the last 10 min of each section. Average peak width, peak amplitude and peak frequency quantifications of islets incubated with 1h thapsigargin, diazoxide and acute thapsigargin+diazoxide at 11mM. **C)** Average peak width, peak amplitude and peak frequency quantifications of islets incubated with 1h thapsigargin, diazoxide and acute thapsigargin+diazoxide at 25mM. Each symbol represents an islet (2-way paired ANOVA, Tukey's correction **P* ≤ .05, ***P* ≤ .01, ****P* ≤ .001, *****P* ≤ .0001, ns, not significant; n=3 WT 71-islets for carbachol, n=3 WT 115-islets for 1h thapsigargin, n=3 WT 45-islets for diazoxide and n=3 WT 58-islets from acute thapsigargin+diazoxide. Data are mean ± SD.)

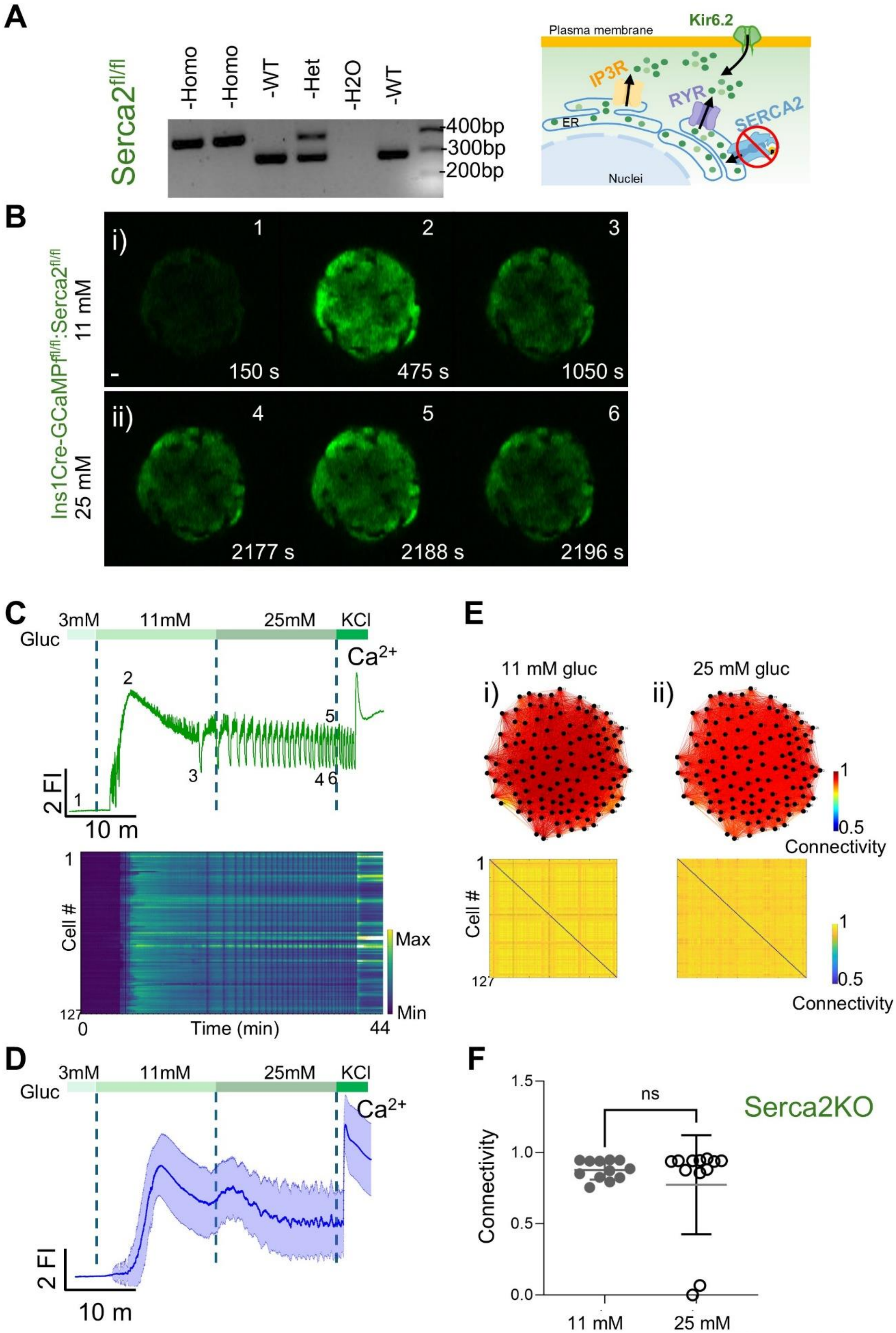
A
Serca2fl/fl
-Homo
-Homo
-WT
-Het
-H2O
-WT
-400bp
-300bp
-200bp
Plasma membrane
Kir6.2
IP3R
RYR
SERCA2
ER
Nuclei
B
Ins1Cre-GCaMPfl/fl:Serca2fl/fl
11 mM
25 mM
i)
ii)
1
2
3
4
5
6
150 s
475 s
1050 s
2177 s
2188 s
2196 s
C
3mM
11mM
25mM
KCl
Gluc
Ca2+
2 FI
10 m
Cell #
Max
Min
Time (min)
0
44
1
127
D
3mM
11mM
25mM
KCl
Gluc
Ca2+
2 FI
10 m
E
11 mM gluc
25 mM gluc
i)
ii)
Connectivity
Cell #
Connectivity
F
ns
Serca2KO
Connectivity
11 mM
25 mM

Supplementary Figure 7. Calcium dynamics in beta-cell-SERCA2A-KO mouse islets. A) Gel showing the specific PCR showing the flox alleles from 2 Serca2$^{fl/fl}$ homozygous, 1 WT, 1 Heterozygous, negative control and DNA ladder. We generated the triple transgenic line Ins1Cre:GCaMPf$^{fl/fl}$:Serca2$^{fl/fl}$, to express the genetically encoded Ca^{2+} indicator GCaMP6 (green), and specifically delete the sarcoendoplasmic reticulum Ca^{2+} ATPase-2 (SERCA2) specifically in pancreatic beta-cells. **B)** Series of confocal calcium imaging of beta-SERCA2-KO islets from showing GCaMP6f signal specifically in beta-cells at the indicated time points (in sec.) during calcium waves oscillations at **i)** 11 mM and **ii)** 25 mM glucose. Scale bar = 25 μm. **C)** Fluorescent trace of the GCaMP6 signal from the islet shown in **(B)** demonstrating calcium influx during the glucose ramp (3 mM, 11 mM, 25 mM) and membrane depolarization induced by 40 mM KCl. The raster plot corresponds to the signal from individual cells. **D)** Average calcium fluorescent traces from islets exposed to a glucose ramp (3 mM, 11 mM, 25 mM) and membrane depolarization by 40 mM KCl (n=3 beta-SERCA2-KO 40-islets). The blue line shows the average GCaMP6f fluorescent and the light-blue the standard deviations from the imaged islets. **E)** Connectivity map and matrix of the islet shown in **(B)** at **i)** 11 mM and **ii)** 25 mM glucose. A connectivity of 1 indicates perfect coordination and 0.5 means that 50% of their active time a cell pair is coordinated. **F)** Connectivity from individual islets n=3 beta-SERCA2-KO 12-islets (unpaired 2-tailed Student's t-test. ns= not significant).

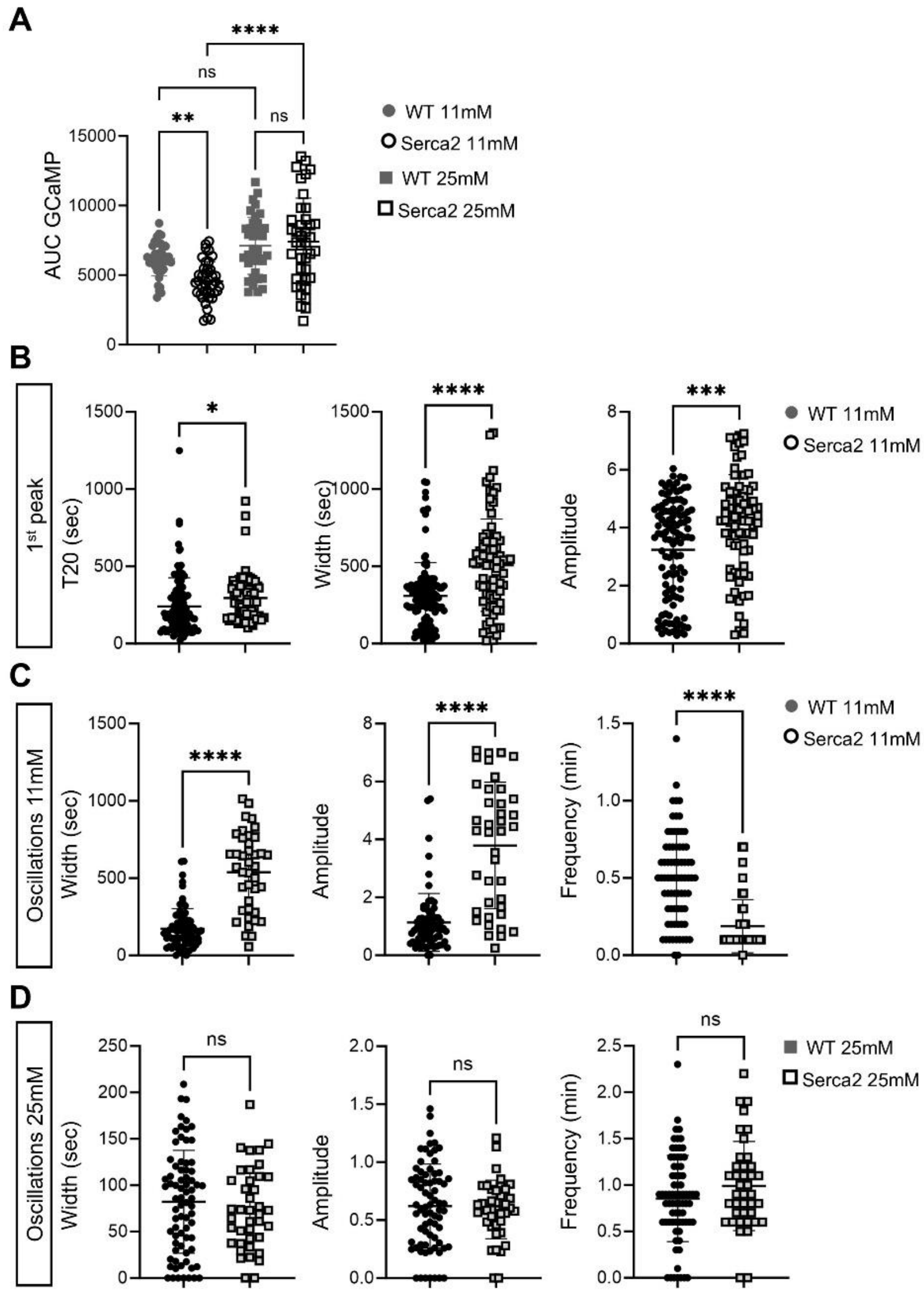

Supplementary Figure 8. beta-cell-SERCA2A-KO mouse islets present altered calcium dynamics. A) Calcium AUC quantifications from WT and beta-SERCA2-KO islets at 11mM and 25mM glucose. **B)** T_{20} is the time taken from the glucose addition in the media to present an increase of 20% over the baseline. The amplitude of the 1st peak is defined as the maximum value recorder during the 1st increase. The peak width is defined as the time spent with an intensity >20% over the base line. The graphs comparing the T_{20}, peak width and peak amplitude between from WT and beta-SERCA2-KO. **C)** The frequency analysis at 11mM and 25mM covering the last 10 min of each section. Average peak width, peak amplitude and peak frequency quantifications of islets from WT and beta-SERCA2-KO at 11mM. **D)** Average peak width, peak amplitude and peak frequency quantifications of islets from WT and beta-SERCA2-KO at 25mM glucose. Each symbol represents an islet (2-way paired ANOVA, Tukey's correction $*P \leq .05$, $**P \leq .01$, $****P \leq .0001$, ns, not significant; n=3 WT 39-islets for glucose and n=3 beta-SERCA2-KO 40-islets. Data are mean ± SD.)

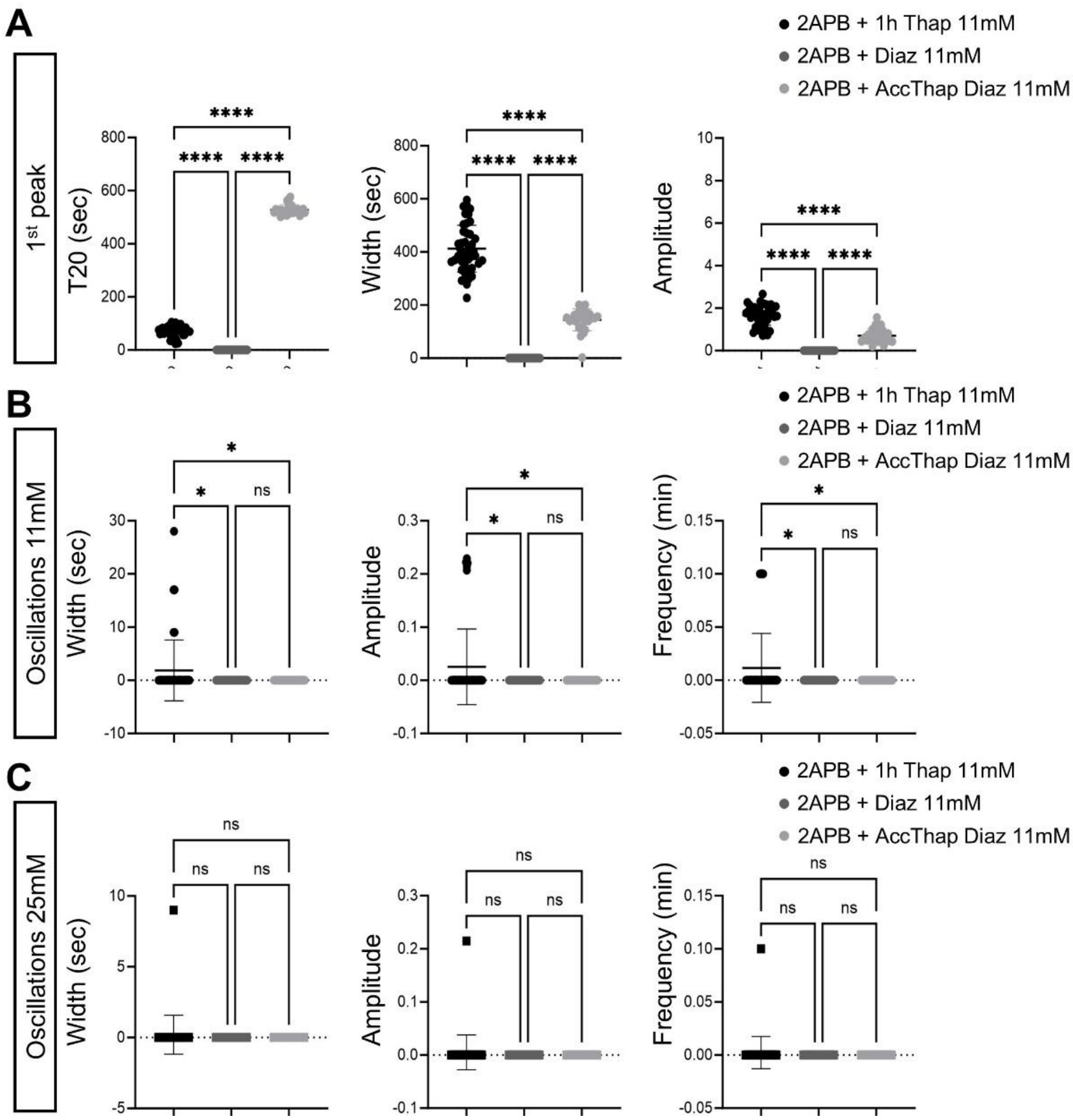

Supplementary Figure 9. Inhibition of IP_3R by 2-APB or ER-calcium stores refilling by thapsigargin display independent effects on islet calcium dynamics. A) The graphs show the T_{20}, peak width and peak amplitude from islets exposed to 1h preincubation thapsigargin and 2-APB, diazoxide + 2-APB, and exposure to diazoxide + 2-APB + thapsigargin delivered after 7.5min of glucose addition. **B)** The frequency analysis at 11mM and 25mM covering the last 10 min of each section. Average peak width, peak amplitude and peak frequency quantifications of islets incubated with 1h thapsigargin and 2-APB, diazoxide + 2-APB, and exposure to diazoxide + 2-APB + thapsigargin delivered after 7.5min of glucose addition. **C)** Average peak width, peak amplitude and peak frequency quantifications of islets incubated after 1h preincubation thapsigargin and 2-APB, diazoxide + 2-APB, and exposure to diazoxide + 2-APB + thapsigargin at 25mM. Each symbol represents an islet (2-way paired ANOVA, Tukey's correction $^*P \leq .05$, $^{**}P \leq .01$, $^{***}P \leq .001$, $^{****}P \leq .0001$, ns, not significant; n=3 WT 43-islets for 1h thapsigargin + 2-APB, n=3 WT 29-islets diazoxide + 2-APB, n=3 WT 26-islets diazoxide + 2-APB + thapsigargin. Data are mean ± SD.)

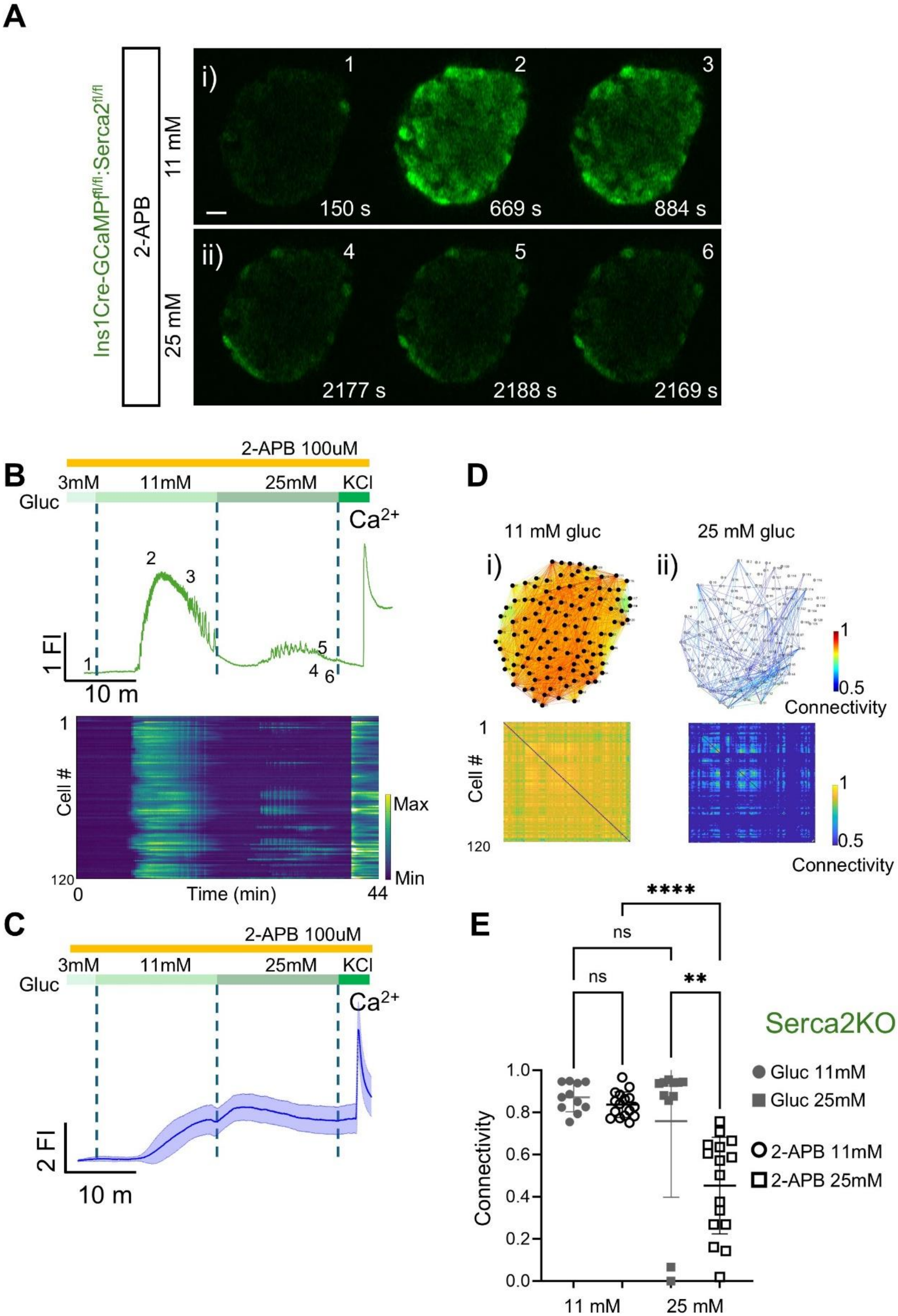
A
Ins1Cre-GCaMP$^{fl/fl}$:Serca2$^{fl/fl}$
2-APB
11 mM
25 mM
i)
ii)
1
2
3
4
5
6
150 s
669 s
884 s
2177 s
2188 s
2169 s
B
2-APB 100uM
3mM
11mM
25mM
KCl
Gluc
Ca^{2+}
1 FI
10 m
Cell #
Max
Min
Time (min)
0
44
120
C
2-APB 100uM
3mM
11mM
25mM
KCl
Gluc
Ca^{2+}
2 FI
10 m
D
11 mM gluc
25 mM gluc
i)
ii)
Connectivity
1
0.5
Cell #
120
E
****
ns
ns
**
Serca2KO
Gluc 11mM
Gluc 25mM
2-APB 11mM
2-APB 25mM
Connectivity
1.0
0.8
0.6
0.4
0.2
0.0
11 mM
25 mM

Supplementary Figure 10. Pharmacological Inhibition of IP_3R by 2-APB in beta-SERCA2-KO Ca^{2+} dynamics and connectivity. A) Series of confocal calcium imaging of beta-SERCA2-KO islet at the indicated time points (in sec.), exposed to the IP3-inhibitor 2-Aminoethoxydiphenyl borate (2-APB) at **i)** 11 mM and **ii)** 25 mM glucose. Scale bar = 25 µm. **B)** Fluorescent trace of the GCaMP6 signal from the islet shown in **(A)** during the glucose ramp (3 mM, 11 mM, 25 mM) and membrane depolarization by 40 mM KCl. The raster plot corresponds to the signal from individual cells. **C)** Average calcium fluorescent traces from islets exposed to 2-APB during a glucose ramp (3 mM, 11 mM, 25 mM) and membrane depolarization by 40 mM KCl. The blue line shows the average GCaMP6f fluorescent and the light-blue the standard deviations from the imaged islets (n=3 beta-SERCA2-KO 40-islets). **D)** Connectivity map and matrix of the islet shown in **(A)** at **i)** 11 mM and **ii)** 25 mM glucose. **E)** Connectivity from individual islets (n=3 beta-SERCA2-KO 11-islets) (unpaired 2-tailed Student's t-test. ns= not significant, ****$P \leq .0001$, **$P \leq .01$).

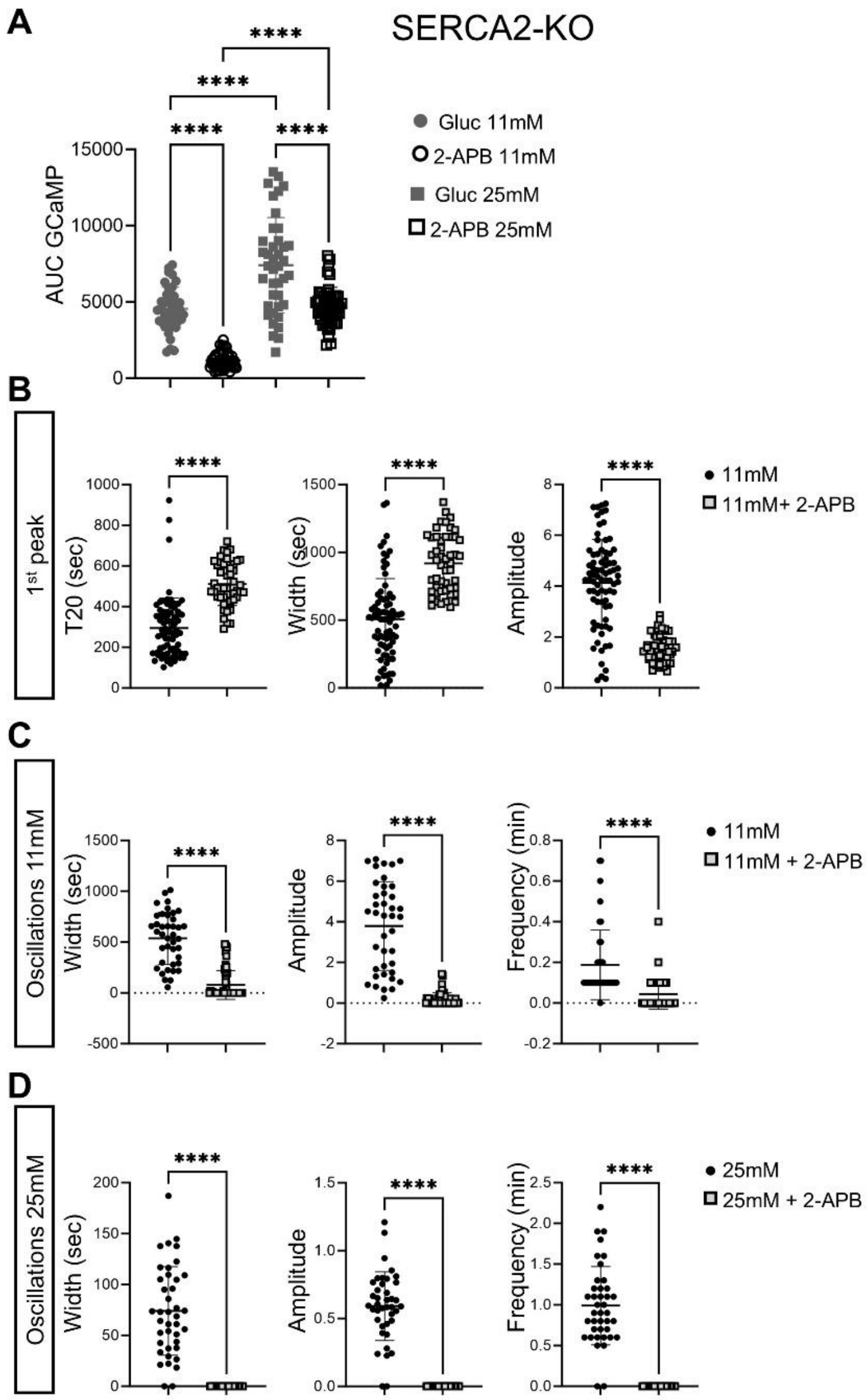

Supplementary Figure 11. IP$_3$R inhibition by 2-APB in beta-SERCA2-KO islet impairs Ca^{2+} dynamics and connectivity. A) Calcium AUC quantifications from glucose and 2-APB (Fig 2) islets at 11mM and 25mM glucose. **B)** T_{20} is the time taken from the glucose addition in the media to present an increase of 20% over the baseline. The amplitude of the 1st peak is defined as the maximum value recorder during the 1st increase. The peak width is defined as the time spent with an intensity >20% over the base line. The graphs comparing the T_{20}, peak width and peak amplitude between glucose and 2-APB exposed beta-SERCA2-KO islets. **C)** The frequency analysis at 11mM and 25mM covering the last 10 min of each section. Average peak width, peak amplitude and peak frequency quantifications of islets incubated with glucose and 2-APB beta-SERCA2-KO islets at 11mM. **D)** Average peak width, peak amplitude and peak frequency quantifications of islets incubated with glucose and 2-APB beta-SERCA2-KO at 25mM. Each symbol represents an islet (2-way paired ANOVA, Tukey's correction **P* ≤ .05, ***P* ≤ .01, *****P* ≤ .0001, ns, not significant; n=3 beta-SERCA2-KO 78-islets for glucose and n=3 beta-SERCA2-KO 50-islets for 2-APB. Data are mean ± SD.)
